# Machine learning models for coagulation dysfunction risk in inpatients administered β-lactam antibiotics

**DOI:** 10.3389/fphar.2024.1503713

**Published:** 2024-11-26

**Authors:** Yuqing Hua, Na Li, Jiahui Lao, Zhaoyang Chen, Shiyu Ma, Xiao Li

**Affiliations:** ^1^ Shandong Engineering and Technology Research Center for Pediatric Drug Development, Shandong Medicine and Health Key Laboratory of Clinical Pharmacy, Department of Clinical Pharmacy, The First Affiliated Hospital of Shandong First Medical University and Shandong Provincial Qianfoshan Hospital, Jinan, China; ^2^ Department of Clinical Pharmacy, Affiliated Hospital of Jining Medical University, Jining, China; ^3^ Center for Big Data Research in Health and Medicine, The First Affiliated Hospital of Shandong First Medical University and Shandong Provincial Qianfoshan Hospital, Jinan, China; ^4^ Ruijin Hospital, Shanghai Jiao Tong University School of Medicine, Shanghai, China

**Keywords:** β-lactam antibiotics, coagulation disorders, risk factors, machine learning, pharmacovigilance

## Abstract

The β-Lactam antibiotics represent a widely used class of antibiotics, yet the latent and often overlooked risk of coagulation dysfunction associated with their use underscores the need for proactive assessment. Machine learning methodologies can offer valuable insights into evaluating the risk of coagulation dysfunction associated with β-lactam antibiotics. This study aims to identify the risk factors associated with coagulation dysfunction related to β-lactam antibiotics and to develop machine learning models for estimating the risk of coagulation dysfunction with real-world data. A retrospective study was performed using machine learning modeling analysis on electronic health record data, employing five distinct machine learning methods. The study focused on adult inpatients discharged from 1 January 2018, to 31 December 2021, at the First Affiliated Hospital of Shandong First Medical University. The models were developed for estimating the risk of coagulation dysfunction associated with various β-lactam antibiotics based on electronic health record feasibility. The dataset was divided into training and test sets to assess model performance using metrics such as total accuracy and area under the curve. The study encompassed risk-factor analysis and machine learning model development for coagulation dysfunction in inpatients administered different β-lactam antibiotics. A total of 45,179 participants were included in the study. The incidence of coagulation disorders related to cefazolin sodium, cefoperazone/sulbactam sodium, cefminol sodium, amoxicillin/sulbactam sodium, and piperacillin/tazobactam sodium was 2.4%, 5.4%, 1.5%, 5.5%, and 4.8%, respectively. Machine learning models for estimating coagulation dysfunction associated with each β-lactam antibiotic underwent validation with 5-fold cross-validation and test sets. On the test set, the optimal models for cefazolin sodium, cefoperazone/sulbactam sodium, cefminol sodium, amoxicillin/sulbactam sodium, and piperacillin/tazobactam sodium yielded AUC values of 0.798, 0.768, 0.919, 0.783, and 0.867, respectively. The study findings suggest that machine learning classifiers can serve as valuable tools for identifying patients at risk of coagulation dysfunction associated with β-lactam antibiotics and intervening based on high-risk predictions. Enhanced access to administrative and clinical data could further enhance the predictive performance of machine learning models, thereby expanding pharmacovigilance efforts.

## Introduction

β-Lactam antibiotics, renowned for their safety, efficacy, and widespread availability (Shenoy et al., 2019), stand as the cornerstone of antimicrobial prescriptions. While generally well-tolerated, a subset of patients encounters diverse adverse reactions following clinical application. Notably, these adverse reactions span a spectrum, including severe renal or hepatic toxicity, neurotoxicity, hemocytopenia, and *Clostridium difficile* infections (Vardakas et al., 2018). A particularly noteworthy adverse effect of this class of drugs is coagulation dysfunction. Drug-Related Coagulation dysfunction (DRCD) is usually characterized by drug-induced prothrombin time prolongation and partial thromboplastin time activation. Over recent decades, global reports have documented instances of bleeding and diminished prothrombin levels associated with β-lactam antibiotics (Nguyen et al., 2015). In 2013, a multicenter clinical study established an association between cefoperazone and coagulopathy (Xin et al., 2013). Subsequently, in January 2014, the China Drug and Food Administration issued a notice regarding serious adverse reactions to cefazolin injections, underscoring the imperative for vigilant monitoring and clinically rational drug use.

Despite a wealth of case reports and small uncontrolled studies on β-lactam antibiotics-related coagulation dysfunction, controlled studies exploring the risks associated with these antibiotics remain limited (Sattler et al., 1988; Wang et al., 2020; Turner et al., 2022; Bai et al., 2023). Given the potential variation in chemical structures among β-lactams, which may influence coagulation dysfunction differently, thorough analyses of individual antibiotics become imperative.

This study aims to address this gap by investigating the incidence and risk factors associated with coagulation dysfunction linked to several β-lactams, including cefazolin sodium, cefoperazone/sulbactam sodium, cefminol sodium, amoxicillin/sulbactam sodium, and piperacillin/tazobactam sodium, utilizing electronic medical record data. Additionally, our objective is to develop machine learning models for estimating the risk of coagulation dysfunction in hospitalized patients receiving these β-lactam antibiotics.

## Materials and methods

### Ethical approval

This study received ethical approval from the Ethics Committee of the First Affiliated Hospital of Shandong First Medical University (Approval No. YXLL-KY-2022-024). The requirement for informed consent was granted a waiver due to the retrospective nature and minimal risk of this study.

### Study design and data source

This retrospective investigation focused on patients discharged between 1 January 2018, and 31 December 2021, who received treatment with cefazolin sodium, cefoperazone/sulbactam sodium, cefminol sodium, amoxicillin/sulbactam sodium, or piperacillin/tazobactam sodium. We utilized clinical data extracted from electronic medical records at the First Affiliated Hospital of Shandong First Medical University. The data for this study was collected from Shandong Provincial Qianfoshan Hospital Healthcare Big Data Platform (SPQHHBDP). The SPQHHBDP integrates multi-source data from hospital information system (HIS), electronic medical records (EMR), laboratory information management system (LIS), picture archiving and communication system (PACS), nursing information system. The encrypted personal identification number was used as a unique identifier to interlink each person’s data information in the above-mentioned database. The patient’s identification number, name, address, and telephone number were encrypted and not accessible to the investigators. Therefore, this study fully adhered to ethical principles and protected patients' privacy.

Data were retrieved from patient medical records via the hospital management systems and healthcare big data platforms. This comprehensive dataset included patient demographics, surgical details, laboratory test results, and diagnostic information, as shown in Supplementary Table S1. For missing data, we employed two strategies: (1) If the proportion of subjects with missing values was less than 5% of the total, those records were removed. (2) For variables with a missing rate below 10%, the missing values were filled using the patient’s information from other hospitalizations within our institution. The dataset was obtained from the hospital’s EHR system, capturing patients from various departments and covering a diverse patient population in terms of age, gender, and clinical diagnoses. To minimize selection bias, random sampling was employed, and bias analysis was conducted to ensure the generalizability of the results.

### Inclusion and exclusion criteria

Inclusion criteria comprised patients with a hospital stay of ≥48 h, aged ≥18 years, and two or more tests for prothrombin time (PT), activated partial thrombin time (APTT), thrombin time (TT), and platelet (PLT) during hospitalization. Exclusion criteria included patients with an unclear route of administration, non-intravenous administration, those receiving heparin, low molecular weight heparin, warfarin, or other anticoagulants, patients lacking baseline parameters or presenting baseline abnormalities, and those with underlying diseases predisposing to coagulation dysfunction.

### Case definition

Baseline values for drug-related coagulation dysfunction (DRCD) definition were determined from the last test before the initial medication use. As shown in Table 1, the baseline abnormalities were defined as TT or PT exceeding the normal upper limit by 3 s or APTT surpassing the normal upper limit by 10 s. Coagulation parameter abnormalities were identified as a 25% increase in APTT, PT, or TT post-medication compared to baseline.

**TABLE 1:** Reference range and definition standards for clinical coagulation parameters.

Coagulation parameters	Reference range	Abnormal baseline	Coagulopathy
APTT(s)	22.7–31.8	>41.8	25% increase compared to baseline
PT(s)	9.8–12.1	>15.1	25% increase compared to baseline
TT(s)	14.0–21.0	>24.0	25% increase compared to baseline
PLT (10^9^/L)	125-350	<100 or >400	25% reduction compared to baseline

### Statistical analysis

Statistical analyses were performed using R software (version 3.6.3) on the healthcare big data platform at the First Affiliated Hospital of Shandong First Medical University. Data with normal distribution were presented as mean ± standard deviation, analyzed by *t*-test or analysis of variance. Non-normally distributed data were expressed as median and interquartile range, assessed by the Wilcoxon rank-sum test. Categorical data were represented as counts or percentages and compared using the Chi-square test. Multivariate logistic regression analysis reported odds ratios (ORs) and 95% confidence intervals (95% CIs). A two-sided *p*-value <0.05 was considered statistically significant.

### Development and validation of machine learning models

Five machine learning methods including logistic regression (LR), random forest (RF), gradient boosting machine (GBM), extreme gradient boosting (XGBoost), and light gradient boosting machine (LightGBM), were employed for predictive model development. The advantages of Logistic Regression are strong explanatory power and high computational efficiency; Random Forest has strong anti-overfitting ability and can perform feature importance evaluation; XGBoost and LightGBM are both improved algorithms based on GBM. XGBoost has better performance, while LightGBM has faster training speed. Therefore, we have selected these five algorithms for the construction of the prediction model and selected the optimal model from them. Patient inclusion involved random division into training and validation sets at a 4:1 ratio. Models underwent 5-fold cross-validation (5-CV) on training sets and independent validation on separate validation sets. To prevent overfitting, univariate analysis screened significantly correlated variables (*p* < 0.05). The features used in the machine learning models were selected based on the results of multivariate logistic regression analysis, where parameters with statistically significant associations (*p* < 0.05) were included. The optimal penalty coefficient (λ) was determined by ten-fold cross-validation, identifying characteristic variables with predictive value. Model performance was evaluated using area under the subject operating characteristic curve (AUC), accuracy (ACC), sensitivity (SEN), and specificity (SPE). ACC gauged overall correctness, while SEN and SPE assessed positive and negative sample prediction accuracy, respectively.

## Results

### Incidence and clinical features of coagulation disorders in patients receiving β-lactam antibiotics

The study analyzed and modeled a cohort of 9,529 participants. The incidence of coagulation dysfunction in patients receiving cefazolin sodium, cefoperazone/sulbactam sodium, cefminox sodium, amoxicillin/sulbactam sodium, and piperacillin/tazobactam sodium was found to be 2.4% (51/2,161), 5.4% (102/1,900), 1.5% (25/1,626), 5.5% (139/2,535), and 4.8% (61/1,282), respectively.

### Analysis of DRCD related factors of study drugs

The study conducted both single-factor (as shown in Supplementary Tables S2–S6) and multi-factor analysis (as shown in Table 2–6) for each β-lactam antibiotic. In the case of cefazolin sodium (CAS), independent factors associated with DRCD included malignant tumors (OR: 4.52, 95% CI 2.000–10.997) and glucocorticoids (OR: 2.172, 95% CI 1.0118–4.716).

**TABLE 2:** Multivariate logistic regression analysis of DRCD occurrence received Cefazolin Sodium.

Factors	*β value*	Wald *value*	*OR value and* 95%*CI*	*p-value*
Daily dose	0.335	0.279	1.398 (0.386∼4.637)	0.597
Time intervals	0.121	1.396	1.129 (0.930∼1.389)	0.237
Total dose	−0.054	0.255	0.947 (0.751∼1.150)	0.613
Malignant tumor	1.509	12.240	4.520 (2.000∼10.997)	<0.001
NASID	−0.360	0.789	0.698 (0.311∼1.538)	0.374
Glucocorticoids	0.776	3.956	2.172 (1.0118∼4.716)	0.047
AEDs	0.199	0.165	1.220 (0.475∼3.272)	0.684
Antipsychotic drug	0.802	1.533	2.440 (0.537∼9.475)	0.216
Antiarrhythmic	0.028	0.004	1.028 (0.411∼2.426)	0.051
Diuretic	0.585	1.677	1.794 (0.742∼4.405)	0.195
*Pseudomonas aeruginosa*	0.732	0.709	2.080 (0.335∼10.837)	0.400
*Streptococcus*	0.708	1.634	2.031 (0.658∼5.913)	0.201
*Acinetobacter* baumannii	0.998	0.668	2.712 (0.122∼22.515)	0.414

**TABLE 3:** Multivariate logistic regression analysis of DRCD occurrence received Cefoperazone.

Factors	*β value*	Wald *value*	*OR value and* 95%*CI*	*p-value*
Gender	0.540	3.524	1.715 (0.987∼3.056)	0.060
Time intervals	0.054	3.820	1.056 (0.998∼1.113)	0.051
Total dose	−0.004	1.310	0.996 (0.988∼1.003)	0.252
Malignant tumor	1.369	18.047	3.932 (2.135∼7.613)	<0.001
Coagulant	0.695	5.338	2.003 (1.110∼3.620)	0.021
AEDs	0.922	9.225	2.515 (1.392∼4.596)	0.002
Antidepressant	18.861	0.000	155381698.928 (0∼ NA)	0.981
Antiarrhythmic	0.478	2.649	1.613 (0.901∼2.859)	0.104
Diuretic	0.592	4.234	1.807 (1.024∼3.173)	0.040

**TABLE 4:** Multivariate logistic regression analysis of DRCD occurrence received cefminox sodium.

Factors	*β value*	Wald *value*	*OR value and* 95%*CI*	*p-value*
Daily dose	−0.720	5.578	0.487 (0.258∼0.865)	0.018
Time intervals	0.187	1.875	1.206 (0.916∼1.576)	0.171
Total dose	0.004	0.009	1.004 (0.911∼1.102)	0.925
Hypertension	0.657	1.667	1.929 (0.722∼5.423)	0.197
Coagulant	1.661	9.314	5.267 (1.774∼15.458)	0.002
NASID	0.362	0.398	1.436 (0.463∼4.471)	0.528
AEDs	1.167	4.143	3.213 (1.046∼10.081)	0.042
Antidepressant	1.343	0.728	3.832 (0.118∼77.164)	0.393
Antianxietic	1.171	4.780	3.225 (1.089∼9.070)	0.029
Diuretic	0.909	3.287	2.483 (0.929∼6.764)	0.070
PPIs	−1.818	5.007	0.162 (0.034∼0.909)	0.025

**TABLE 5:** Multivariate logistic regression analysis of DRCD occurrence received sulbactam sodium.

Factors	*β value*	Wald *value*	*OR value and* 95%*CI*	*p-value*
Age	0.009	2.368	1.009 (0.998∼1.021)	0.124
Time intervals	0.172	15.901	1.187 (1.090∼1.291)	<0.001
Total dose	0.001	0.059	1.001 (0.991∼1.011)	0.808
Pneumonia	0.331	0.937	1.392 (0.699∼2.685)	0.333
Coagulant	0.770	4.737	2.159 (1.052∼4.239)	0.030
Contrast medium	1.751	13.413	5.763 (2.206∼14.607)	<0.001
*Pseudomonas aeruginosa*	−0.319	0.161	0.727 (0.108∼2.819)	0.688
*Acinetobacter* baumannii	0.013	0.000	1.013 (0.114∼6.029)	0.989
*Klebsiella pneumoniae*	0.220	0.186	1.246 (0.423∼3.185)	0.666
Surgery	2.487	3.811	12.029 (1.053∼274.422)	0.051

**TABLE 6:** Multivariate logistic regression analysis of DRCD occurrence received Mezlocillin Sodium and Sulbactam

Factors	*β value*	Wald *value*	*OR value and* 95%*CI*	*p-value*
Time intervals	−0.04389	0.48434	0.957 (0.804∼1.0391)	0.486
Total dose	0.00781	1.76286	1.008 (0.997∼1.022)	0.184
CKD	0.58574	1.3441	1.796 (0.632∼4.671)	0.246
Hypertension	0.67792	2.97254	1.970 (0.911∼4.290)	0.085
Pneumonia	−0.06716	0.02435	0.935 (0.392∼2.141)	0.876
Coagulant	1.47466	28.11951	4.370 (2.537∼7.579)	<0.001
AEDs	1.7169	13.33576	5.567 (2.297∼14.792)	<0.001
Antipsychotic drug	0.25341	0.06096	1.288 (0.141∼8.951)	0.805
Antianxietic	0.76216	2.84677	2.143 (0.863∼5.134)	0.092
Antiarrhythmic	−0.02856	0.00473	0.972 (0.424∼2.178)	0.945
Diuretic	0.74388	2.75662	2.104 (0.874∼5.111)	0.097

For cefoperazone/sulbactam sodium (CSSS), independent factors for DRCD were malignant tumors (OR: 3.932, 95% CI 2.135–7.613), anticoagulants (OR: 2.00, 95% CI 1.110–3.620), antiepileptic drugs (OR: 2.515, 95% CI 1.392–4.596), and diuretics (OR: 1.807, 95% CI 1.024–3.173).

The risk factors for DRCD associated with cefminox sodium (CMS) included daily dose (OR: 0.487, 95% CI 0.258–0.865), anticoagulants (OR: 5.267, 95% CI 1.774–15.458), antiepileptics (OR: 3.213, 95% CI 1.046–10.081), anxiolytics (OR: 3.225, 95% CI 1.089–9.070), and proton pump inhibitors (OR: 0.162, 95% CI 0.034–0.909).

Mezlocillin/sulbactam sodium (MMS) DRCD independent risk factors comprised drug use time (OR: 1.187, 95% CI 1.090–1.291), contrast agents (OR: 5.763, 95% CI 2.206–14.607), and coagulants (OR: 2.159, 95% CI 1.052–4.239).

Piperacillin/tazobactam sodium (PTS) independent risk factors for DRCD were anticoagulants (OR: 4.370, 95% CI 2.537–7.579) and antiepileptic drugs (OR: 5.567, 95% CI 2.297–14.792).

### Validation and evaluation of DRCD prediction models for study drugs

**FIGURE 1:**
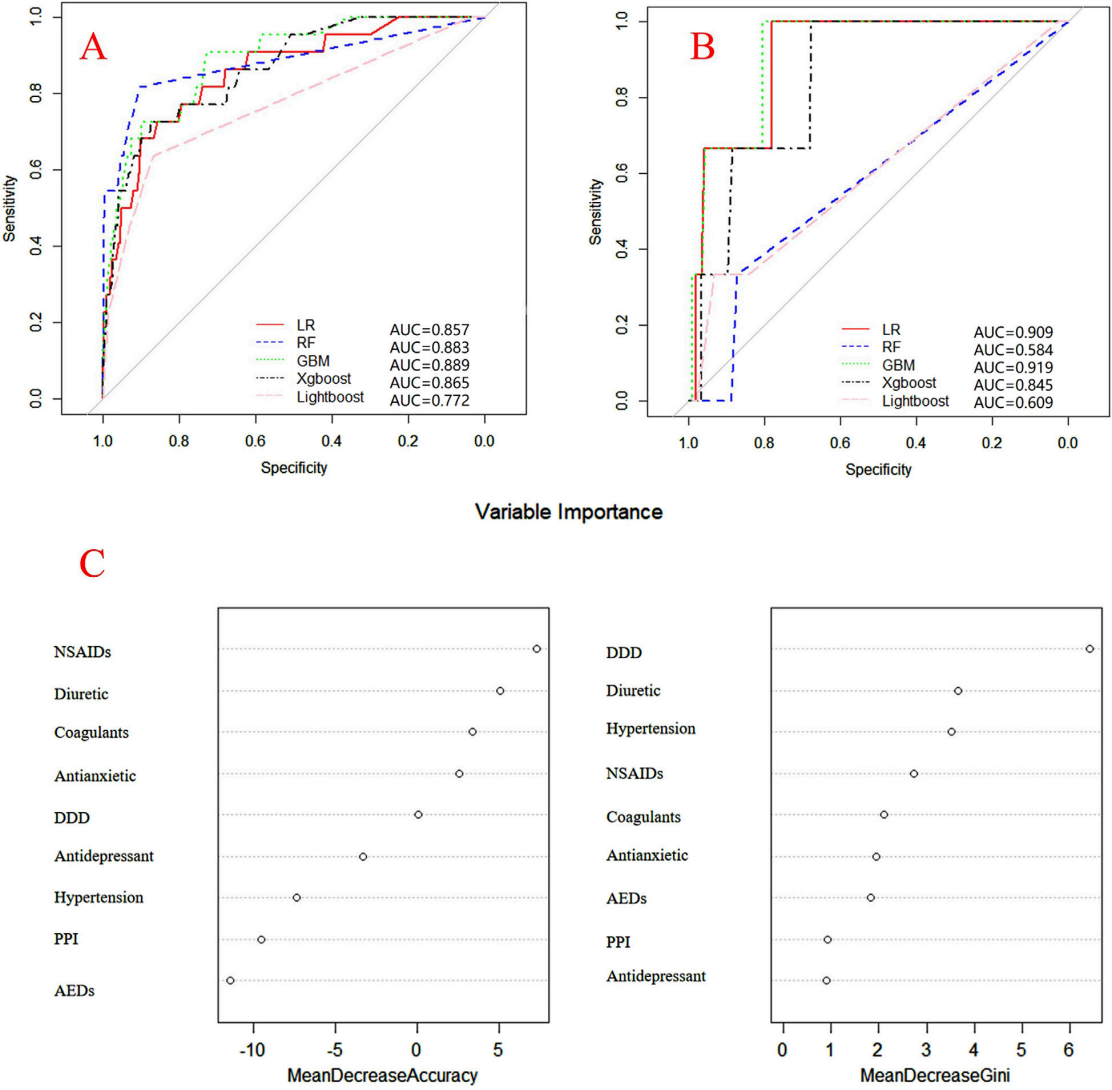
ROC curves of models for cefazolin sodium associated DRCD and the results of the importance of related factors. **(A)** internal validation; **(B)** external validation; **(C)** analysis on the importance of DRCD related factors of cefazolin sodium.

**FIGURE 2:**
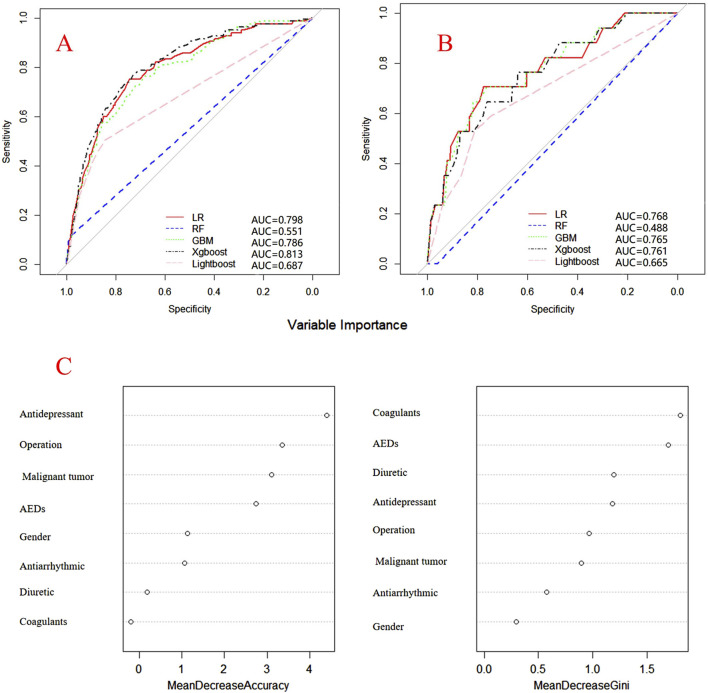
ROC curves of models for cefoperazone associated DRCD and the results of the importance of related factors. **(A)** internal validation; **(B)** external validation; **(C)** analysis on the importance of DRCD related factors of cefoperazone.

**FIGURE 3:**
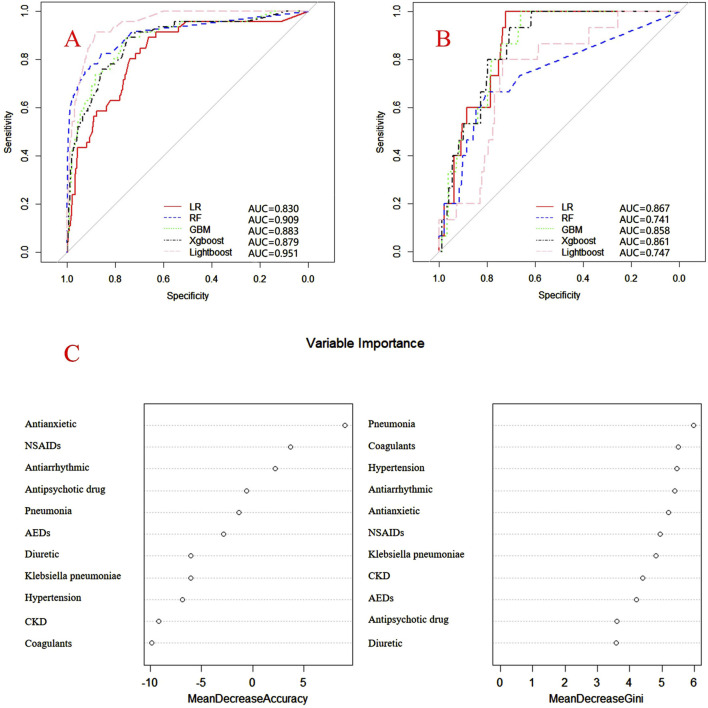
ROC curves of models for cefminox sodium associated DRCD and the results of the importance of related factors. **(A)** internal validation; **(B)** external validation; **(C)** analysis on the importance of DRCD related factors of cefminox sodium.

**FIGURE 4:**
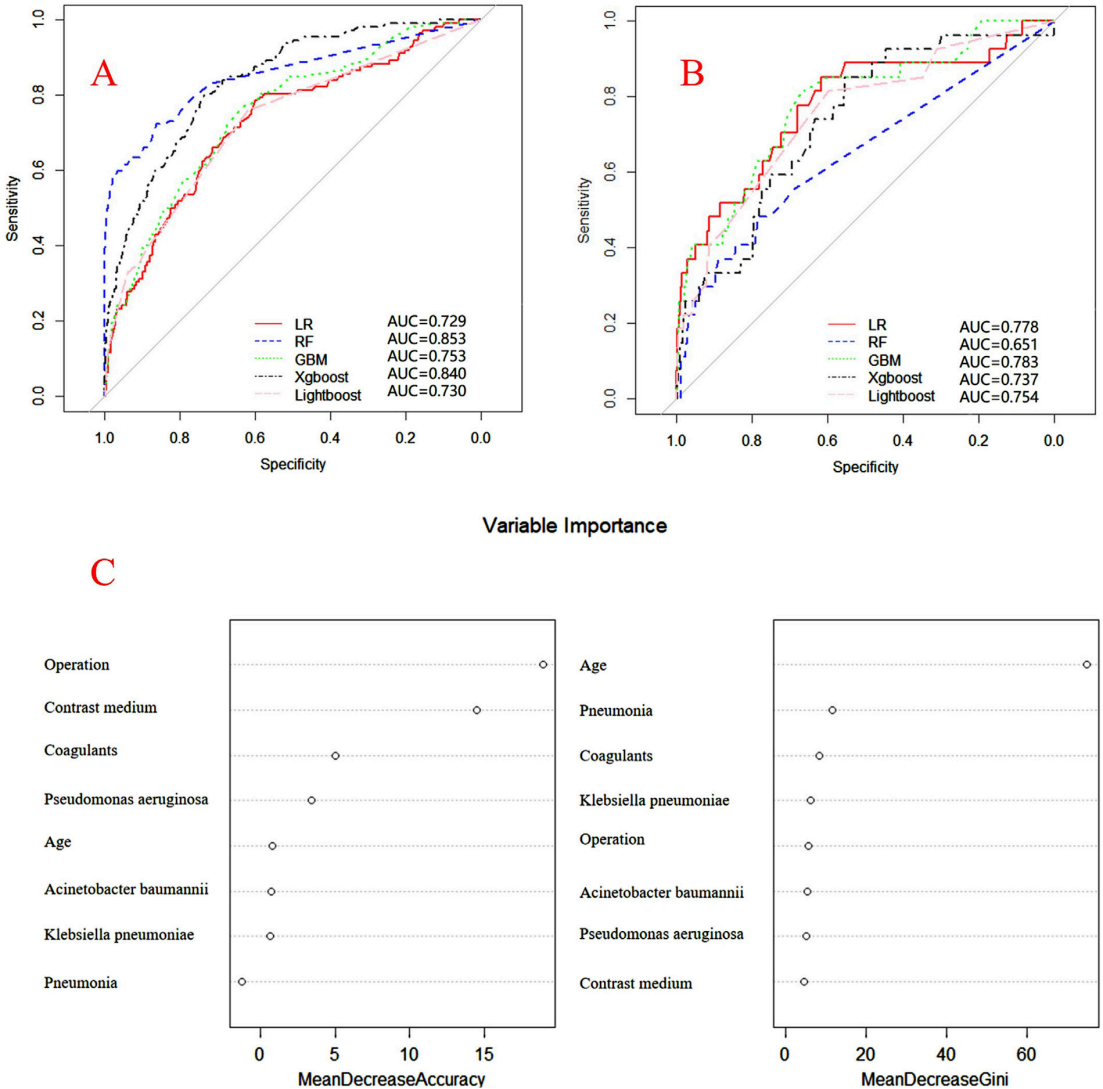
ROC curves of models for mezlocillin associated DRCD and the results of the importance of related factors. **(A)** internal validation; **(B)** external validation; **(C)** analysis on the importance of DRCD related factors of mezlocillin.

**FIGURE 5:**
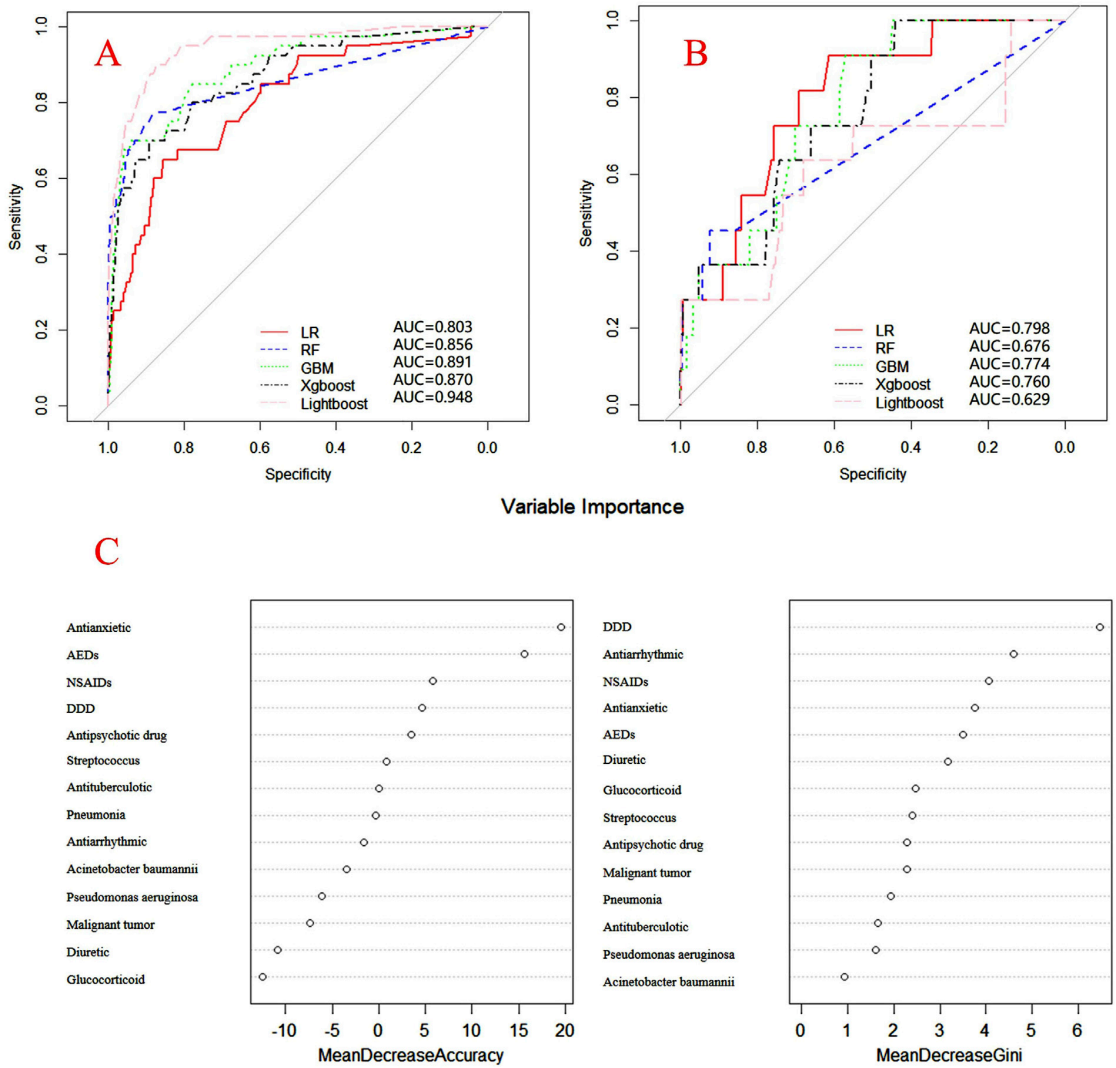
ROC curves of models for piperacillin associated DRCD and the results of the importance of related factors. **(A)** internal validation; **(B)** external validation; **(C)** analysis on the importance of DRCD related factors of piperacillin.

Internal and external verification results for each β-lactam antibiotic revealed optimal AUC and ACC values, as shown in Figures 1–5. For CAS, the AUC was 0.798. CSSS had an AUC of 0.768, CMS 0.919, MMS 0.783, and PTS 0.867. Validation results illustrated the robustness of the prediction models, with key factors impacting DRCD risk elucidated through ROC curve analyses.

For CAS, factors such as daily dose, non-steroidal anti-inflammatory drugs, anxiolytics, antiepileptics, antipsychotics, and antiarrhythmic drugs were identified as highly correlated with DRCD in cefazolin sodium. For CSSS, major factors affecting the DRCD risk model of cefoperazone sodium and sulbactam sodium included surgery, antiepileptic drugs, antidepressants, diuretics, and malignant tumors. Similarly, for CMS, significant factors impacting the occurrence of DRCD in cefminox sodium included non-steroidal anti-inflammatory drugs, daily dose, diuretics, anxiolytics, and hypertension. In MMS, factors such as age, surgery, contrast agent, pneumonia, procoagulants, and others were found to have a considerable impact on the production of DRCD of mezlocillin/sulbactam sodium. For PTS, the occurrence of DRCD in injectable piperacillin/tazobactam was significantly influenced by risk factors such as anxiolytics, non-steroidal anti-inflammatory drugs, antiarrhythmic drugs, pneumonia, anticoagulants, and hypertension.

The predictive models can be integrated into clinical decision support systems to identify patients at high risk of DRCD, enabling timely intervention and personalized medication adjustments, ultimately improving patient outcomes.

## Discussion

The emergence of coagulation disorders related to β-lactam antibiotics poses a multifaceted challenge, necessitating a nuanced exploration of contributing factors. Our study delves into the intricate web of associations, shedding light on the complexity of determinants influencing DRCD in this antibiotic class. The random forest algorithm assesses the importance of variables through two primary methods: Mean Decrease in Gini and Mean Decrease in Accuracy. These two methods determine the importance of variables by calculating the impact of each factor on the impurity of all decision tree nodes in the forest, as well as the impact of randomly adding noise to a certain feature on the accuracy of the model. The larger the value, the more important the variable.

### Molecular mechanisms and patient demographics

Cephalosporin-induced DRCD can be attributed to the molecular structure containing the N-methylthiotetrazolium side (NMTT), directly impacting vitamin K participation in carboxylation reactions and heightening the risk of bleeding (Lipsky, 1983). In contrast, penicillin-related DRCD may result from penicillin binding to platelet surface proteins, rendering them antigenic and triggering an immune response, leading to decreased platelet count and DRCD (Chong et al., 2013). The stronger association between cefminox and piperacillin with coagulation dysfunction may be related to their specific molecular structures that affect clotting pathways. For instance, cefminox has been associated with reduced prothrombin activity, leading to increased risk of coagulation disorders (Wu et al., 2022).

Analyzing patient demographics, age emerges as a contributing factor to DRCD related to β-lactam antibiotics, with a higher incidence in the elderly. Age-related factors such as diminished immunity, organ aging, poor renal excretion, and drug accumulation elevate the risk of adverse reactions. Thus, caution is warranted to ensure medication safety for the elderly (Fu and Perloff, 2022). Furthermore, our findings underscore a positive correlation between total dosage and DRCD risk, emphasizing the importance of adhering to recommended antibiotic doses in clinical applications (Rochoy et al., 2022).

### Gender disparities and underlying diseases

Gender appears to influence DRCD occurrence, particularly evident in a higher likelihood among men receiving cefoperazone injection. These gender-related differences may be linked to body fat composition and hydrophilic drug combinations, suggesting the necessity for personalized clinical guidance (Karamian et al., 2022).

Patients with underlying diseases, particularly those with malignant tumors, exhibited a significantly higher incidence of DRCD. This association might be linked to circulating tumor cells affecting the coagulation system, extending APTT, TT, or PT times, and elevating DRCD risk (Ward et al., 2021). Chronic kidney disease patients, particularly in the study of piperacillin/tazobactam sodium for injection, demonstrated a heightened susceptibility to DRCD, possibly due to alterations in coagulation system interactions under metabolic conditions in renal failure, as suggested by studies on the impact of microparticles on coagulation (Lutz et al., 2014).

### Unexpected findings and medication interactions

Contrary to general expectations, our study revealed higher DRCD incidence in hypertensive patients treated with cefminox sodium for injection and piperacillin for injection. This unexpected finding prompts further investigation to explore potential connections between hypertension and DRCD, especially in the context of diuretic use (Antza et al., 2018; Kearsley and Stocks, 2021).

Analyzing concurrent medications, anticoagulant use was associated with an increased DRCD risk, seemingly contradictory to the expected therapeutic effects. However, this association was likely influenced by the inclusion of patients undergoing surgeries that required anticoagulants. Similarly, non-steroidal anti-inflammatory drugs exhibited a significant impact, affecting COX-1 activity and transiently influencing platelet function (Scharf, 2012).

### Glucocorticoids and protective effects

Inconsistencies arose in the relationship between glucocorticoid use and DRCD, with our study indicating a higher incidence, contrary to previous research (Graversen et al., 2012; Coelho et al., 2015; Isidori et al., 2015). This unexpected outcome suggests the need for a more nuanced exploration of the potential liver-related effects of glucocorticoids on DRCD occurrence.

Surprisingly, the inclusion of proton pump inhibitors appeared to inhibit DRCD occurrence, suggesting a potential protective effect. This aligns with their clinical use in alleviating gastrointestinal bleeding, underscoring the importance of considering the comprehensive medication profile (Lanas et al., 2018).

### Bacterial associations and surgery

Examining bacterial associations, *Streptococcus* and Gram-negative bacteria, including *Pseudomonas aeruginosa*, *Acinetobacter* baumannii, and *Klebsiella pneumoniae*, were linked to increased DRCD risk. The interaction between these bacteria and the coagulation system components was identified as a potential contributor to DRCD (Herwald et al., 2003; Bergmann and Hammerschmidt, 2007).

Surgery emerged as a significant related factor, supporting existing research on postoperative patients being more prone to DRCD (Ranucci, 2015; Bolliger and Tanaka, 2017). The local trauma and mechanical bleeding associated with surgery might contribute to DRCD, necessitating vigilant monitoring during antibiotic administration in surgical settings.

### Limitations

One of the limitations of this study is its retrospective design, which limits the ability to infer causal relationships between β-lactam antibiotic use and coagulation dysfunction. Additionally, since the data were collected from a single hospital, the generalizability of the findings to other populations or healthcare settings may be limited. Future studies should aim to validate the model on external datasets from different regions or healthcare systems to enhance its applicability.

## Conclusion

In conclusion, our study unravels the intricate interplay of factors influencing DRCD in β-lactam antibiotics. Individualized clinical medication plans, considering patient demographics, comorbidities, and concurrent medications, are pivotal to mitigate the risk of DRCD. Further research is imperative to delve into nuanced relationships and unexpected outcomes, providing a more comprehensive understanding of DRCD determinants.

## Data Availability

The raw data supporting the conclusions of this article will be made available by the authors, without undue reservation.
