# Supplementary material for: Machine learning models for coagulation dysfunction risk in inpatients administered β-lactam antibiotics
Source: Front Pharmacol. 2024 Nov 26;15:1503713. doi: 10.3389/fphar.2024.1503713 (PMC11628276; doi:10.3389/fphar.2024.1503713)
Supplement: Supplementary file 1 [file DataSheet1.docx]

**Supporting Information**

**Machine Learning Models for Coagulation Dysfunction Risk in Inpatients Administered β-Lactam Antibiotics**

Yuqing Hua^1,2#^, Na Li^1#^, Jiahui Lao^3#^, Zhaoyang Chen^1^, Shiyu Ma^4^, Xiao Li^1^*

1. *Shandong Engineering and Technology Research Center for Pediatric Drug Development, Shandong Medicine and Health Key Laboratory of Clinical Pharmacy, Department of Clinical Pharmacy,* *The First Affiliated Hospital of* *Shandong First Medical University & Shandong Provincial Qianfoshan Hospital, Jinan 250014, China*
2. *Department of Clinical Pharmacy, Affiliated Hospital of Jining Medical University*
3. *Center for Big Data Research in Health and Medicine, The First Affiliated Hospital of Shandong First Medical University & Shandong Provincial Qianfoshan Hospital, Jinan 250014, China*
4. *Ruijin Hospital, Shanghai Jiao Tong University School of Medicine, Shanghai 200025, China*

#These authors contributed equally to this work.

* To whom correspondence should be addressed.

**Corresponding author**

**Xiao Li**

E-mail: [lixiao1688@163.com](mailto:lixiao1688@163.com) or [x.li@sdu.edu.cn](mailto:x.li@sdu.edu.cn)

ORCID id: 0000-0002-1148-9898

**Supplemental Table**

Table S1. Inpatient indicators extracted from the electronic medical record system for analysis

| Demographics | Age、Youth、Middle-aged、The old、Gender、BMI |
| --- | --- |
| Dose | Daily dose、Time intervals、Total dose |
| Comorbidities | Renal failure、Malignant tumor、Chronic kidney disease(CKD)、Hypertension、Diabetes、Chronic cholecystitis、Chronic cholecystitis、Hypothyroidism、chronic obstructive pulmonary disease(COPD)、Pneumonia、Acidosis |
| Concomitant medication | Coagulant、NASID、Glucocorticoids、AEDs、Antipsychotic drug、Antidepressant、Antianxietic、Antithyroid drugs、Antiarrhythmic、Antituberculotic、AMs、Antineoplastic、Diuretic、PPIs、Contrast medium、Microbe、Pseudomonas aeruginosa、Streptococcus、Acinetobacter baumannii、Klebsiella pneumoniae |
| Surgery |  |

Table S2. Single factor analysis results of cefazolin sodium associated DRCD

| Factors | DRCD(51) | Non-DRCD(2110) | All(2161) | *χ^2^* | *P value* |
| --- | --- | --- | --- | --- | --- |
| Demographics |  |  |  |  |  |
| Age(years) | 57[49.5,66.5] | 58[49,67] |  | 54712.5 | 0.837 |
| Youth | 7(13.73%) | 380(18.01%) | 387(17.91%) | 0.62 | 0.430 |
| Middle-aged | 33(64.71%) | 1356(64.27%) | 1389(64.28%) | 0.004 | 0.948 |
| The old | 11(21.57%) | 374(17.73%) | 385(17.82%) | 0.50 | 0.478 |
| Gender |  |  |  | 1.14 | 0.285 |
| male | 27(52.94%) | 958(45.4%) | 985(45.58%) |  |  |
| Female | 24(47.06%) | 1152(54.6%) | 1176(54.42%) |  |  |
| BMI | 24.6[23.03,26.95] | 25.23[22.60,27.64] |  | 36627.5 | 0.490 |
| Dose |  |  |  |  |  |
| Daily dose | 1.06[0.53,1.06] | 0.53[0.53,1.06] |  | 46105 | 0.046 |
| Time intervals | 3[2,4] | 2[1,4] |  | 11011 | <0.001 |
| Total dose | 2.12[1.06,4.24] | 1.06[0.53,3.18] |  | 11420.5 | 0.002 |
| Comorbidities |  |  |  |  |  |
| Renal failure | 0(0%) | 2(0.09%) | 0(0.09%) | 0.05 | 0.826 |
| Malignant tumor | 31(60.78%) | 653(30.95%) | 684(31.65%) | 20.50 | <0.001 |
| Chronic kidney disease(CKD) | 2(3.92%) | 127(6.02%) | 129(5.97%) | 0.39 | 0.532 |
| Hypertension | 19(37.25%) | 657(31.14%) | 676(31.28%) | 0.87 | 0.352 |
| Diabetes | 4(7.84%) | 205(9.72%) | 209(9.67%) | 0.20 | 0.655 |
| Chronic cholecystitis | 2(3.92%) | 64(3.03%) | 66(3.05%) | 0.13 | 0.716 |
| Hypothyroidism | 1(1.96%) | 10(0.47%) | 11(0.51%) | 2.17 | 0.140 |
| (COPD) | 0(0%) | 2(0.09%) | 0(0.09%) | 0.05 | 0.826 |
| Pneumonia | 6(11.76%) | 103(4.88%) | 109(5.04%) | 4.93 | 0.0265 |
| Acidosis | 0 | 6(0.28%) | 6(0.28%) | 0.15 | 0.703 |
| Concomitant medication |  |  |  |  |  |
| Coagulant | 30(58.82%) | 1152(54.6%) | 1182(54.7%) | 0.36 | 0.549 |
| NASID | 21(41.18%) | 1404(66.54%) | 1425(65.94%) | 14.26 | <0.001 |
| Glucocorticoids | 31(60.78%) | 504(23.89%) | 535(24.76%) | 36.39 | <0.001 |
| AEDs | 37(72.55%) | 910(43.13%) | 947(43.82%) | 17.51 | <0.001 |
| Antipsychotic drug | 7(13.73%) | 30(1.42%) | 37(1.71%) | 44.79 | <0.001 |
| Antidepressant | 0(0%) | 2(0.09%) | 2(0.09%) | 0.05 | 0.826 |
| Antianxietic | 9(17.65%) | 176(8.34%) | 185(8.56%) | 5.51 | 0.019 |
| Antithyroid drugs | 0(0%) | 1(0.05%) | 1(0.05%) | 0.02 | 0.876 |
| Antiarrhythmic | 19(37.25%) | 382(18.1%) | 401(18.56%) | 12.08 | <0.001 |
| Antituberculotic | 1(1.96%) | 0(0%) | 1(0.05%) | 41.39 | <0.001 |
| AMs | 0(0%) | 3(0.14%) | 3(0.14%) | 0.07 | 0.788 |
| Antineoplastic | 2(3.92%) | 22(1.04%) | 24(1.11%) | 3.76 | 0.053 |
| Diuretic | 29(56.86%) | 503(23.84%) | 532(24.62%) | 29.26 | <0.001 |
| PPIs | 41(80.39%) | 1797(85.17%) | 1838(85.05%) | 0.89 | 0.345 |
| Contrast medium | 2(3.92%) | 43(2.04%) | 45(2.08%) | 0.87 | 0.352 |
| Microbe |  |  |  |  |  |
| Pseudomonas aeruginosa | 5(9.8%) | 28(1.33%) | 33(1.53%) | 23.80 | <0.001 |
| Streptococcus | 11(21.57%) | 203(9.62%) | 214(9.9%) | 7.97 | 0.00476 |
| Acinetobacter baumannii | 2(3.92%) | 13(0.62%) | 15(0.69%) | 7.89 | 0.005 |
| Klebsiella pneumoniae | 4(7.84%) | 78(3.7%) | 82(3.79%) | 2.35 | 0.126 |
| Surgery | 51(100%) | 2092(99.15%) | 2143(99.17%) | 0.44 | 0.508 |

Table S3. Single Factor Analysis Results of Cefoperazone associated DRCD

| Factors | DRCD(102) | Non-DRCD(1798) | All(1900) | *χ^2^* | *P value* |
| --- | --- | --- | --- | --- | --- |
| Demographics |  |  |  |  |  |
| Age(years) | 58.5[50,68.75] | 61[50,70] |  | 95107.5 | 0.527 |
| Youth | 12(11.76%) | 308(17.13%) | 320(16.84%) | 1.98 | 0.159 |
| Middle-aged | 65(63.73%) | 997(55.45%) | 1062(55.89%) | 2.68 | 0.102 |
| The old | 25(24.51%) | 493(27.42%) | 518(27.26%) | 0.41 | 0.521 |
| Gender |  |  |  | 3.89 | 0.048 |
| male | 68(66.67%) | 1020(56.73%) | 1088(57.26%) |  |  |
| Female | 34(33.33%) | 778(43.27) | 812(42.74%) |  |  |
| BMI | 23.5[21.50,25.37] | 23.18[20.76,25.95] |  | 49926.5 | 0.555 |
| Dose |  |  |  |  |  |
| Daily dose | 1.5[3,6] | 6[3,6] |  | 100347 | 0.082 |
| Time intervals | 2[1,5] | 1[1,4] |  | 31506.5 | <0.001 |
| Total dose | 9[6,24] | 6[6,18] |  | 35128 | 0.013 |
| Comorbidities |  |  |  |  |  |
| Renal failure | 0 | 1(0.06%) | 1(0.05%) | 0.06 | 0.812 |
| Malignant tumor | 74(72.55%) | 931(51.78%) | 1055(52.89%) | 16.71 | <0.001 |
| Chronic kidney disease(CKD) | 6(5.88%) | 248(13.79%) | 254(13.37%) | 5.22 | 0.022 |
| Hypertension | 30(29.41%) | 555(30.87%) | 585(30.79%) | 0.10 | 0.757 |
| Diabetes | 23(22.55%) | 274(15.24%) | 297(15.63%) | 3.91 | 0.048 |
| Chronic cholecystitis | 33(32.25%) | 543(30.2%) | 576(30.32%) | 0.21 | 0.645 |
| Hypothyroidism | 1(0.98%) | 16(0.89%) | 17(0.89%) | 0.01 | 0.925 |
| (COPD) | 0 | 21(1.17%) | 21(1.11%) | 1.20 | 0.272 |
| Pneumonia | 14(13.73%) | 238(13.24%) | 252(13.26%) | 0.02 | 0.887 |
| Acidosis | 3(2.94%) | 18(1%) | 21(1.11%) | 3.32 | 0.068 |
| Concomitant medication |  |  |  |  |  |
| Coagulant | 56(54.9%) | 412(22.91%) | 468(24.63%) | 53.20 | <0.001 |
| NASID | 54(52.94%) | 745(41.43%) | 799(42.05%) | 5.24 | 0.022 |
| Glucocorticoids | 51(50%) | 743(41.32%) | 794(41.79%) | 2.99 | 0.084 |
| AEDs | 64(62.75%) | 573(31.87%) | 637(33.53%) | 41.30 | <0.001 |
| Antipsychotic drug | 5(4.9%) | 53(2.95%) | 58(3.05%) | 1.25 | 0.264 |
| Antidepressant | 3(2.94%) | 6(0.33%) | 9(0.47%) | 13.92 | <0.001 |
| Antianxietic | 14(13.73%) | 243(13.52%) | 257(13.75%) | 0 | 0.952 |
| Antithyroid drugs | 0 | 3(0.17%) | 3(0.16%) | 0.17 | 0.680 |
| Antiarrhythmic | 47(46.08%) | 549(30.53%) | 596(31.37%) | 10.83 | <0.001 |
| Antituberculotic | 0 | 3(0.17%) | 3(0.16%) | 0.17 | 0.680 |
| AMs | 0 | 5(0.28%) | 5(0.26%) | 0.28 | 0.594 |
| Antineoplastic | 6(5.88%) | 61(3.39%) | 67(3.53%) | 1.76 | 0.185 |
| Diuretic | 56(54.9%) | 589(32.76%) | 645(33.95%) | 21.11 | <0.001 |
| PPIs | 92(90.2%) | 1608(89.43%) | 1700(89.47%) | 0.06 | 0.807 |
| Contrast medium | 8(7.84%) | 122(6.79%) | 130(6.84%) | 0.17 | 0.681 |
| Microbe |  |  |  |  |  |
| Pseudomonas aeruginosa | 6(5.88%) | 64(3.56%) | 70(3.68%) | 1.47 | 0.226 |
| Streptococcus | 24(23.53%) | 437(24.3%) | 461(24.26%) | 0.03 | 0.859 |
| Acinetobacter baumannii | 1(0.98%) | 32(1.78%) | 33(1.74%) | 0.36 | 0.548 |
| Klebsiella pneumoniae | 10(9.8%) | 163(9.07%) | 173(9.11%) | 0.06 | 0.801 |
| Surgery | 96(94.12%) | 1291(71.8%) | 1387(73%) | 0.44 | <0.001 |

Table S4. Single factor analysis results of cefminox sodium associated DRCD

| Factors | DRCD(25) | Non-DRCD(1626) | All(1651) | *χ^2^* | *P value* |
| --- | --- | --- | --- | --- | --- |
| Demographics |  |  |  |  |  |
| Age(years) | 66[55,72] | 63[51,72] |  | 19420.5 | 0.702 |
| Youth | 3(12%) | 279(17.16%) | 282(17.08%) | 0.46 | 0.496 |
| Middle-aged | 14(56%) | 856(52.64%) | 870(52.7%) | 0.11 | 0.739 |
| The old | 8(32%) | 491(30.2%) | 499(30.22%) | 0.038 | 0.846 |
| Gender |  |  |  | 1.20 | 0.273 |
| male | 17(68%) | 928(57.07%) | 945(57.24%) |  |  |
| Female | 8(32%) | 698(42.93%) | 706(42.76%) |  |  |
| BMI | 25.5[23.28,26.41] | 23.51[21.09,25.95] |  | 8403.5 | 0.081 |
| Dose |  |  |  |  |  |
| Daily dose | 2[1.5,3] | 3[2,4] |  | 27748 | 0.001 |
| Time intervals | 4[2,7] | 1[1,3] |  | 4401 | <0.001 |
| Total dose | 9[4.5,16] | 4[3,6] |  | 6229.5 | 0.001 |
| Comorbidities |  |  |  |  |  |
| Renal failure | 0 | 1(0.06%) | 1(0.06%) | 0.02 | 0.901 |
| Malignant tumor | 16(64%) | 911(56.03%) | 927(56.15%) | 0.64 | 0.425 |
| Chronic kidney disease(CKD) | 3(12%) | 127(7.81%) | 130(7.87%) | 0.60 | 0.440 |
| Hypertension | 16(64%) | 566(34.81%) | 582(35.25%) | 9.20 | 0.002 |
| Diabetes | 5(20%) | 248(15.25%) | 253(15.32%) | 0.43 | 0.513 |
| Chronic cholecystitis | 5(20%) | 239(14.7%) | 244(14.78%) | 0.55 | 0.459 |
| Hypothyroidism | 1(4%) | 15(0.92%) | 16(0.97%) | 2.43 | 0.119 |
| (COPD) | 0 | 20(1.23%) | 20(1.21%) | 0.31 | 0.557 |
| Pneumonia | 5(20%) | 213(13.1%) | 218(13.2%) | 1.02 | 0.312 |
| Acidosis | 0 | 12(0.74%) | 12(0.73%) | 0.19 | 0.666 |
| Concomitant medication |  |  |  |  |  |
| Coagulant | 8(32%) | 153(9.41%) | 161(9.75%) | 14.28 | <0.001 |
| NASID | 14(56%) | 317(19.5%) | 331(20.05%) | 20.47 | <0.001 |
| Glucocorticoids | 11(44%) | 565(34.75%) | 576(34.89%) | 0.93 | 0.335 |
| AEDs | 11(44%) | 170(10.46%) | 181(10.96%) | 28.38 | <0.001 |
| Antipsychotic drug | 2(8%) | 22(1.35%) | 24(1.45%) | 7.59 | 0.006 |
| Antidepressant | 1(4%) | 3(0.18%) | 4(0.24%) | 14.83 | <0.001 |
| Antianxietic | 9(36%) | 153(9.41%) | 162(9.81%) | 19.67 | <0.001 |
| Antithyroid drugs | 0 | 6(0.37%) | 6(0.36%) | 0.09 | 0.761 |
| Antiarrhythmic | 7(28%) | 321(19.74%) | 328(19.87%) | 1.05 | 0.304 |
| AMs | 0 | 3(0.18%) | 0(0.18%) | 0.05 | 0.830 |
| Antineoplastic | 0 | 51(3.14%) | 51(3.09%) | 0.81 | 0.368 |
| Diuretic | 13(52%) | 349(21.46%) | 362(21.93%) | 13.41 | <0.001 |
| PPIs | 22(88%) | 1561(96%) | 1583(95.88%) | 3.99 | 0.046 |
| Contrast medium | 0 | 38(2.34%) | 38(2.3%) | 0.60 | 0.439 |
| Microbe |  |  |  |  |  |
| Pseudomonas aeruginosa | 1(4%) | 39(2.4%) | 40(2.42%) | 0.27 | 0.605 |
| Streptococcus | 9(36%) | 363(22.32%) | 372(22.53%) | 2.64 | 0.104 |
| Acinetobacter baumannii | 0 | 13(0.8%) | 13(0.79%) | 0.20 | 0.654 |
| Klebsiella pneumoniae | 0 | 89(5.47%) | 89(5.39%) | 1.45 | 0.229 |
| Surgery | 21(84%) | 1175(72.26%) | 1196(72.44%) | 1.70 | 0.192 |

Table S5. Single factor analysis results of mezlocillin sodium and sulbactam sodium associated DRCD

| Factors | DRCD(139) | Non-DRCD(2396) | All(2535) | *χ^2^* | *P value* |
| --- | --- | --- | --- | --- | --- |
| Demographics |  |  |  |  |  |
| Age(years) | 68[60,75.5] | 64[51,74] |  | 145252 | 0.011 |
| Youth | 16(11.51%) | 428(17.86%) | 444(17.51%) | 3.67 | 0.055 |
| Middle-aged | 62(44.6%) | 1124(46.91%) | 1186(46.79%) | 0.28 | 0.596 |
| The old | 61(43.88%) | 844(35.23%) | 905(35.7%) | 4.29 | 0.038 |
| Gender |  |  |  | 0.35 | 0.555 |
| male | 93(66.91%) | 1544(64.44%) | 1637(64.58%) |  |  |
| Female | 46(33.09%) | 852(35.56%) | 898(35.42%) |  |  |
| BMI | 24.44[26.99,21.30] | 24.22[26.64,21.48] |  | 44070 | 0.913 |
| Dose |  |  |  |  |  |
| Daily dose | 7.5[3.75,7.5] | 7.5[3.75,7.5] |  | 161708 | 0.532 |
| Time intervals | 4[2,8] | 1[1,4] |  | 46058 | <0.001 |
| Total dose | 30[11.25,52.5] | 7.5[7.5,22.5] |  | 51766.5 | <0.001 |
| Comorbidities |  |  |  |  |  |
| Renal failure | 1(0.72%) | 5(0.21%) | 6(0.24%) | 1.45 | 0.228 |
| Malignant tumor | 46(33.09%) | 759(31.68%) | 805(31.76%) | 0.12 | 0.727 |
| Chronic kidney disease(CKD) | 20(14.39%) | 349(14.57%) | 369(14.56%) | 0 | 0.954 |
| Hypertension | 67(48.2%) | 964(40.23%) | 1031(40.67%) | 3.46 | 0.063 |
| Diabetes | 31(22.3%) | 417(17.4%) | 448(17.67%) | 2.17 | 0.141 |
| Chronic cholecystitis | 9(6.47%) | 197(8.22%) | 206(8.13%) | 0.54 | 0.464 |
| Hypothyroidism | 1(0.72%) | 17(071%) | 18(0.71%) | 0 | 0.989 |
| COPD | 6(4.32%) | 137(5.72%) | 143(5.64%) | 0.48 | 0.486 |
| Pneumonia | 49(35.25%) | 617(25.75%) | 666(26.27%) | 6.12 | 0.013 |
| Acidosis | 4(2.88%) | 17(0.71%) | 21(0.83%) | 7.52 | 0.006 |
| Concomitant medication |  |  |  |  |  |
| Coagulant | 32(23.02%) | 246(10.27%) | 278(10.97%) | 21.89 | <0.001 |
| NASID | 0 | 31(1.29%) | 31(1.22%) | 1.82 | 0.177 |
| Glucocorticoids | 0 | 39(1.63%) | 39(1.54%) | 2.30 | 0.130 |
| AEDs | 0 | 25(1.04%) | 25(0.99%) | 1.46 | 0.226 |
| Antipsychotic drug | 0 | 3(0.13%) | 3(0.12) | 0.17 | 0.676 |
| Antianxietic | 0 | 8(0.33%) | 8(0.32%) | 0.47 | 0.495 |
| Antithyroid drugs | 0 | 1(0.04%) | 1(0.04%) | 0.06 | 0.810 |
| Antiarrhythmic | 0 | 32(1.34%) | 32(1.26%) | 1.88 | 0.170 |
| Diuretic | 0 | 32(1.34%) | 32(1.26%) | 1.88 | 0.170 |
| PPIs | 0 | 60(2.5%) | 60(2.37%) | 3.57 | 0.059 |
| Contrast medium | 15(10.79%) | 66(2.75%) | 81(3.2%) | 27.43 | <0.001 |
| Microbe |  |  |  |  |  |
| Pseudomonas aeruginosa | 18(12.95%) | 123(5.13%) | 141(5.56%) | 15.28 | <0.001 |
| Streptococcus | 58(41.73%) | 1016(42.4%) | 1074(42.37%) | 0.02 | 0.875 |
| Acinetobacter baumannii | 23(16.55%) | 72(3.01%) | 95(3.75%) | 66.79 | <0.001 |
| Klebsiella pneumoniae | 36(25.9%) | 227(9.47%) | 263(10.37%) | 38.12 | <0.001 |
| Surgery | 117(84.17%) | 1508(62.94%) | 1625(64.1%) | 25.74 | <0.001 |

Table S6. Single factor analysis results of mezlocillin sodium and sulbactam sodium associated DRCD

| Factors | DRCD(61) | Non-DRCD(1221) | All(1282) | *χ^2^* | *P value* |
| --- | --- | --- | --- | --- | --- |
| Demographics |  |  |  |  |  |
| Age(years) | 63[53,73] | 63[52,70] |  | 35810.5 | 0.612’ |
| Youth | 8(13.11%) | 190(15.56%) | 198(15.44%) | 0.27 | 0.606 |
| Middle-aged | 34(55.74%) | 688(56.35%) | 722(56.32%) | 0.009 | 0.925 |
| The old | 19(31.15%) | 343(28.09%) | 362(28.24%) | 0.27 | 0.605 |
| Gender |  |  |  |  |  |
| male | 41(67.21%) | 796(65.19%) | 837(65.29%) | 0.10 | 0.746 |
| Female | 20(32.79%) | 425(34.81%) | 445(34.71%) |  |  |
| BMI | 23.4[20.80,25.95] | 23.44[20.90,26.04] |  | 22108.5 | 0.884 |
| Dose |  |  |  |  |  |
| Daily dose | 13.5[4.5,13.5] | 9[4.5,13.5] |  | 35732.5 | 0.570 |
| Time intervals | 3[2,4] | 1[1,4] |  | 12231 | <0.001 |
| Total dose | 22.5[13.5,45] | 13.5[5,9,40] |  | 13176.5 | 0.002 |
| Comorbidities |  |  |  |  |  |
| Malignant tumor | 30(49.18%) | 644(52.74%) | 674(52.57%) | 0.30 | 0.586 |
| Chronic kidney disease(CKD) | 13(21.31%) | 134(10.97%) | 147(11.47%) | 6.12 | 0.013 |
| Hypertension | 32(52.46%) | 396(32.43%) | 428(33.39%) | 10.48 | 0.001 |
| Diabetes | 14(22.95%) | 194(15.89%) | 208(16.22%) | 2.13 | 0.144 |
| Chronic cholecystitis | 14(22.95%) | 291(23.83%) | 305(23.79%) | 0.02 | 0.875 |
| Hypothyroidism | 1(1.64%) | 13(1.06%) | 14(1.09%) | 0.18 | 0.673 |
| (COPD) | 0(0%) | 41(3.36%) | 41(3.2%) | 2.12 | 0.146 |
| Pneumonia | 26(42.62%) | 336(27.52%) | 362(28.24%) | 6.54 | 0.010 |
| Acidosis | 1(1.64%) | 12(0.98%) | 13(1.01%) | 0.25 | 0.617 |
| Concomitant medication |  |  |  |  |  |
| Coagulant | 33(54.1%) | 248(20.31%) | 281(21.92%) | 38.75 | <0.001 |
| NASID | 31(50.82%) | 465(38.08%) | 496(38.69%) | 3.97% | 0.046 |
| Glucocorticoids | 29(47.54%) | 643(52.66%) | 672(52.42%) | 0.61 | 0.434 |
| AEDs | 45(73.77%) | 362(29.65%) | 407(31.75%) | 52.20 | <0.001 |
| Antipsychotic drug | 7(11.48%) | 30(2.46%) | 37(2.89%) | 16.86 | <0.001 |
| Antidepressant | 0(0%) | 7(0.57%) | 7(0.55%) | 0.35 | 0.553 |
| Antianxietic | 16(26.23%) | 125(10.24%) | 141(11%) | 15.18 | <0.001 |
| Antithyroid drugs | 0(0%) | 3(0.25%) | 3(0.23%) | 0.15 | 0.698 |
| Antiarrhythmic | 33(54.1%) | 387(31.7%) | 420(32.76%) | 13.24 | <0.001 |
| AMs | 0 | 5(0.41%) | 5(0.39%) | 0.25 | 0.617 |
| Antineoplastic | 1(1.64%) | 26(2.13%) | 27(2.11%) | 0.07 | 0.795 |
| Diuretic | 44(72.13%) | 372(30.47%) | 416(32.45%) | 46.01 | <0.001 |
| PPIs | 47(77.05%) | 996(81.57%) | 1043(81.36%) | 0.78 | 0.376 |
| Contrast medium | 2(3.28%) | 72(5.9%) | 74(5.77%) | 0.73 | 0.392 |
| Microbe |  |  |  |  |  |
| Pseudomonas aeruginosa | 4(6.56%) | 67(5.49%) | 71(5.54%) | 0.13 | 0.721 |
| Streptococcus | 22(36.07%) | 424(34.73%) | 446(34.79%) | 0.05 | 0.830 |
| Acinetobacter baumannii | 2(3.28%) | 30(2.46%) | 32(2.5%) | 0.16 | 0.688 |
| Klebsiella pneumoniae | 15(24.59%) | 144(11.79%) | 159(12.4%) | 8.76 | 0.003 |
| Surgery | 52(85.25%) | 845(69.21%) | 897(69.97%) | 7.11 | 0.008 |
